# Radiographic outcome over 15 years in patients with early rheumatoid arthritis – a BARFOT-study

**DOI:** 10.1186/s41927-026-00660-w

**Published:** 2026-05-27

**Authors:** Maria L. E. Andersson, Ingäld Hafström, Kristina Forslind

**Affiliations:** 1 Department of Clinical Sciences Lund, Rheumatology, Faculty of Medicine, Lund University, Lund, Sweden; 2 Spenshult Research and Development Centre, Bäckagårdsvägen 47 , Halmstad, 302 74 Sweden; 3 Department of Environmental and Biosciences School of Business, Innovation and Sustainability, Halmstad University, Halmstad, Sweden; 4 Division of Gastroenterology and Rheumatology, Department of Medicine Huddinge, Karolinska Institute, Stockholm, Sweden; 5 Karolinska University Hospital, Stockholm, Sweden

**Keywords:** Rheumatoid arthritis, Radiography, Erosions, Joint space narrowing, Progression

## Abstract

**Objectives:**

To investigate the progression of radiographic damage in hands and feet of patients with early rheumatoid arthritis monitored prospectively for 15 years, and to search for predictors.

**Methods:**

This study comprises 990 patients from the BARFOT cohort, included 1992–2006. Radiographs of the hands and feet were performed over time and evaluated by the Sharp van der Heijde scoring (SHS) method. The patients were divided into three clusters, using progression over 15 years of erosion score and joint space narrowing score. The cluster analyses were conducted using K-means cluster analysis. Disease activity was measured by Disease Activity Score 28 (DAS28) and erythrocyte sedimentation rate (ESR), and physical function by Health Assessment Questionnaire (HAQ).

**Results:**

Cluster 1 consisted of 54, cluster 2 of 258 and cluster 3 of 678 patients. Of the patients with the highest radiographic progress during the 15 years (cluster 1), 59% were included in the 1990s, and they were overall more often seropositive (94%), and had at baseline higher SHS, and lower DAS28. Of the patients with the smallest radiographic progression (cluster 3), 45% were included in the 1990s, and they were overall less often seropositive, had at baseline lower SHS, higher DAS28 and higher tender joint count (TJC). At baseline, there was no difference in the choice of treatment between the clusters. From the two-year follow-up, a higher proportion of patients in cluster 3 were untreated. Seropositivity and less tender joints predicted belonging to cluster 1, whereas besides seropositivity also older age, female sex, and erosions at baseline predicted belonging to cluster 2.

The worse radiographic progression in cluster 1 and 2 was associated with higher DAS28 throughout the 15 years, and with worse HAQ at both 8 and 15 years, compared with cluster 3.

**Conclusions:**

This study highlights the presence of distinct subgroups of rheumatoid arthritis, which points to the importance of early and individualised treatment strategies.

**Supplementary Information:**

The online version contains supplementary material available at 10.1186/s41927-026-00660-w.

## Introduction

Rheumatoid arthritis (RA) is a chronic inflammatory disease characterised by symmetrical peripheral polyarthritis, which may seriously affect the quality of life for patients.

Imaging evaluation in RA has evolved significantly in last decades and even if the modern imaging technics like ultrasound and magnetic resonance imaging have unequivocal place in the assessment of early disease (especially for synovial inflammation, and bone oedema), radiographic evaluation of hands and feet is still the most widely used imaging technic for quantification of joint damage in RA [1].

The commonly used assessment methods for radiographs of hands and feet in studies of RA focus on bone and cartilage damage, erosions and joint space narrowing respectively. Conventional radiographic bone erosion of periarticular cortical bone remains one of the hallmarks of RA, is relevant for diagnosis, treatment and monitoring of the disease [1, 2].

The nature and progression of joint damage differ significantly between patients, ranging from erosion-free disease, non-progression of existing erosion to progression of different magnitudes. Radiographic progression is not always associated with clinically severe signs and symptoms [3].

Several studies as well as reports on actively treated patients in clinical care have indicated that early, active, and continuous intervention with disease modifying anti-rheumatic drug (DMARD) therapy prevents or slows down the radiographic damage in early RA [4, 5]. Furthermore, biologic DMARDs (bDMARDs) together with or without methotrexate have been shown to slow down the radiographic progression more than conventional triple therapy [6–8].

In chronic conditions, long-term outcomes confirm the effectiveness of outpatient rheumatology care [9]. Improved long-term outcomes have also been reported in clinical care, including good clinical status [5], remission rates [10], declining need for joint replacement surgery [11], and good survival in patients who respond to conventional DMARDs (cDMARD) [12]. Recently, studies showed that treat-to-target were superior to conventional treatment in terms of achieving clinical remission [13, 14].

In the current treatment paradigm joint damage is no longer clearly a direct consequence of disease activity (by index measures) for all patients, and radiologic progression may remain in those patients with erosive disease at diagnosis, although in clinical remission [15].

In the present study, the aims were to investigate radiographic progression, in hands and feet, in patients with early RA monitored prospectively for 15 years in outpatient rheumatology units, and to investigate the predictive factors.

## Patients and methods

### Patients

The “BARFOT” (Better Anti Rheumatic FarmacO Therapy) study involves six rheumatology units from southern Sweden covering a population of 1.5 million [9, 16]. BARFOT is an observational study of patients with recent onset of RA (disease duration ≤ 1 year) [17] fulfilling the 1987 ACR classification criteria [18], and included 1992–2006. At the follow-up after 15 years, 1634 patients participated, of which 990 had radiographs performed at least at baseline and at the 15-year follow-up.

The patients were assessed at baseline and at predefined follow-up visits. At baseline, patient characteristics such as age, gender, smoking habits (current, previous or never smokers), menopausal age, comorbidities, medical history and symptom duration were registered. The patients were started with DMARDs in accordance with the recommended treatment strategy in Sweden. All data were registered in the BARFOT database.

### Radiographic assessments

Posterior–anterior radiographs of the hands and feet were assessed at baseline and at 1, 2, 5, 8 and 15 years according to the van der Heijde modification of the Sharp score (SHS) where 32 joints in the hands and 12 in the feet are assessed, calculating total SHS (range 0–448), erosion score (ES) (range 0–280), and joint space narrowing score (JSN) (range 0–168) [19]. Erosive disease was defined as the presence of erosions on radiographs of the hands, wrists or feet at inclusion. Erosions were defined as presence of a cortical break on the radiographs. Radiographic progress is defined as SHS ≥ 3 units per year [20] and rapid radiographic progression (RRP) of joint damage as SHS ≥ 5 units per year [21].

The films were read by one of two experienced readers. Double readings of a fraction of films showed good agreement between the two readers. The intraclass correlation coefficient for SHS was excellent (0.940–0.998).

### Clinical disease assessments

At baseline and at 1, 2, 5, 8 and 15 years disease activity was assessed by the composite index Disease Activity Score calculated on 28 joints (DAS28; range 0–9.4, best to worst) [22]. DAS28 includes the number of swollen joints counts (SJC) (range 0–28), number of tender joints counts (TJC) (range 0 28), patient’s global assessment of health (PatGA) measured on a visual analogue scale (VAS) (ranged 0–100 mm, best to worse) and the erythrocyte sedimentation rate (ESR; 0–150 mm/h). Pain was assessed by a VAS (0–100 mm), and rheumatoid factor (RF) was measured according to the current laboratory standards at the participating hospitals. Antibodies to cyclic citrullinated peptides (ACPA) were detected using the ELISA CCP2test (Euro-Diagnostica, Malmö, Sweden) or the BioPlex 2200 Anti-CCP kit (Bio-Rad Laboratories, CA, USA). Seropositivity was defined as RF and/or ACPA positive. The Swedish version of the Stanford Health Assessment Questionnaire (HAQ) was used to measure daily life function (range 0–3, best to worst) [23].

### Statistical analysis

The analyses were performed using IBM SPSS Statistics (version 29).

K-means cluster analysis was conducted to identify distinct subgroups of patients based on longitudinal changes in erosion score (ES) and joint space narrowing (JSN) over the 15-year follow-up period. Prior to clustering, both variables were standardised (z-scores) to ensure comparability and to prevent disproportionate influence of scale differences on cluster formation. Clustering was performed using the k-means algorithm with Euclidean distance as the similarity metric and random initial centroid seeds. The algorithm iteratively reassigned cases to clusters to minimise within-cluster sum of squares and maximise between-cluster separation, until convergence criteria were met (no further changes in cluster membership and minimal change in cluster centres). To determine the optimal number of clusters, solutions ranging from two to six clusters were evaluated. Model selection was guided by cluster interpretability, clinical relevance of the resulting profiles, and relative separation between clusters [24, 25]. This approach enabled the identification of three clinically meaningful radiographic progression patterns, supporting a characterisation of long-term joint damage trajectories within the cohort, Fig. S1.

The three clusters were compared using clinical data. The clinical data were not normally distributed, Shapiro-Wilk *p* < 0.05. To test the differences between the three groups, the Kruskal-Wallis test was used; if *p* < 0.05, pairwise comparisons were performed using the Mann-Whitney U test. The chi-squared test was used for nominal and ordinal data. The missing data were not replaced. Logistic regression models were performed with cluster 3 as a reference to study predictors for cluster 1 and 2.

The significance tests were two-tailed and conducted at the 0.05 level of significance. Statistical analyses were performed using SPSS version 29.0 statistical software (IBM Corp., Armonk, NY, USA).

## Results

Demographic and disease characteristics at baseline of the 990 patients with radiographs at 15 years are shown in Table 1. The patients were more frequently women (71%), median age 54 years and symptom duration of 6 months, 71% were seropositive. Three clusters were identified: cluster 1 consisted of 54, cluster 2 of 258 and cluster 3 of 678 patients, being seropositive in 94, 84 and 64%, respectively. The patients with the smallest radiographic progression during the 15 years (cluster 3) were younger (53 years), had the lowest SHS, were less often seropositive and had higher TJC. At baseline 95% of the patients in cluster 3 started with glucocorticoids (GC) and/ or DMARD, and the corresponding figures for cluster 1 and 2 were 88% and 93%, respectively.

Of the 990 patients, 451 (46%) were included in the 1990s. In cluster 1, 32 (59%), in cluster 2, 116 (45%) and in cluster 3, 303 (45%). 539 patients were included in the 2000s, 22 (41%) in cluster 1, 142 (55%) in cluster 2 and 375 (55%) in cluster 3, *p* = 0.115. According to the protocol, radiography was meant to be performed at six occasions. In cluster 1 and 2, 68%, and in cluster 3, 69% had radiographs at all six occasions, and 95%, 99% and 98% respectively for cluster 1, 2 and 3 had radiographs from four occasions. There was an even distribution of women and men in the clusters, 72%, 75% and 70% women in cluster 1, 2, and 3, respectively.

**Table 1:** Descriptives at baseline for the 990 patients with radiographs over 15 years, divided into three clusters

*N*	All Median (IQR)	Cluster 1 Median (IQR)	Cluster 2 Median (IQR)	Cluster 3 Median (IQR)	*P*-overall	Post hoc
	990	54	258	678		
Age	54 (18)	56 (12)	56 (14)	53 (20)	< 0.001	Cl.1 vs. 3 *p* = 0.045; Cl. 2 vs. 3 *p* < 0.001
Included 1990s, n (%)	452 (46)	32 (59)	116 (45)	303 (45)	0.115	
Sex female, n (%)	707 (71)	39 (72)	194 (75)	473 (70)	0.257	
Smoking						
Never smoker, n (%)	393 (40)	18 (33)	97 (38)	277 (41)		
Current smoker, n (%)	268 (27)	17 (32)	76 (29)	175 (26)	0.651	
Previous smoker, n (%)	328 (33)	19 (35)	85 (33)	224 (33)		
RF-positive, n (%)	640 (65)	45 (83)	199 (77)	396 (58)	< 0.001	
ACPA positive, n (%)	505 (62)	33 (85)	145 (81)	327 (55)	< 0.001	
Seropos, n (%)	701 (71)	51 (94)	216 (84)	433 (64)	< 0.001	
Disease duration, months	6 (5)	6 (5)	6 (4)	6 (5)	0.587	
ES	0 (1.0)	0.5 (4.0)	1.0 (3.3)	0 (0)	< 0.001	Cl.1 vs. 3 *p* < 0.001 Cl. 2 vs. 3 *p* < 0.001
JSN	0 (3.0)	0 (6.3)	1.0 (6.3)	0 (2.0)	< 0.001	Cl.1 vs. 3 *p* = 0.033 Cl. 2 vs. 3 *p* < 0.001
SHS	0 (6.0)	4.0 (9.3)	3.0 (10.3)	0 (3.0)	< 0.001	Cl.1 vs. 3 *p* < 0.001 Cl. 2 vs. 3 *p* < 0.001
DAS28	5.30 (1.62)	4.75 (1.37)	5.32 (1.84)	5.35 (1.62)	0.012	Cl. 1 vs. 2 *p* = 0.035 Cl. 1 vs. 3 *p* < 0.001
DAS28(3)	5.23 (1.54)	4.71 (1.33)	5.20 (1.64)	5.30 (1.52)	0.014	Cl.1 vs. 2 *p* = 0.036 Cl.1 vs. 3 *p* = 0.010
TJC (0–28)	7 (9)	3 (5)	6 (8)	8 (9)	0.001	Cl. 1 vs. 2 *p* = 0.002 Cl. 1 vs. 3 *p* < 0.001 Cl. 2 vs. 3 *p* = 0.008
SJC (0–28)	10 (8)	9 (7)	10 (8)	10 (8)	0.640	
ESR (mm/h)	29 (29)	30 (25)	32 (30)	28 (29)	0.015	Cl. 2 vs. 3 *p* = 0.021
Pain (0-100)	48 (36)	50 (47)	47 (37)	48 (39)	0.476	
PatGA (0-100)	47 (39)	44 (37)	46 (32)	47 (41)	0.450	
HAQ (0–3)	0.88 (0.88)	1.00 (0.62)	0.88 (0.78)	0.88 (0.88)	0.841	
Treatment						
No, n (%)	54 (5)	6 (11)	16 (6)	32 (5)	0.045	
GC, n (%)	38 (4)	5 (9)	10 (4)	23 (3)		
MTX	534 (54)	31 (57)	148 (57)	355 (52)		
Other cDMARD, n (%)	358 (36)	12 (22)	83 (32)	262 (39)		
bDMARD, n (%)	7 (1)	0 (0)	1 (0)	6 (1)		

ACPA, Anti-citrullinated protein antibodies; bDMARD, biological disease modifying anti-rheumatic drug; cDMARD, conventional DMARD; DAS28, 28 joints-disease activity score; DAS28(3), DAS28 excluding PatGA; ES, erosion score; ESR, erythrocyte sedimentation rate; GC, glucocorticoids; HAQ, health assessment questionnaire; JSN, joint space narrowing score; MTX, methotrexate; PatGA, patient global assessment; RF, rheumatoid factor; Seropos, ACPA and/or RF positive; SJC, 28-joints swollen joint count; SHS, Sharp van der Heijde score; TJC, 28-joints tender joint count

### Radiographic progression

**Fig. 1:**
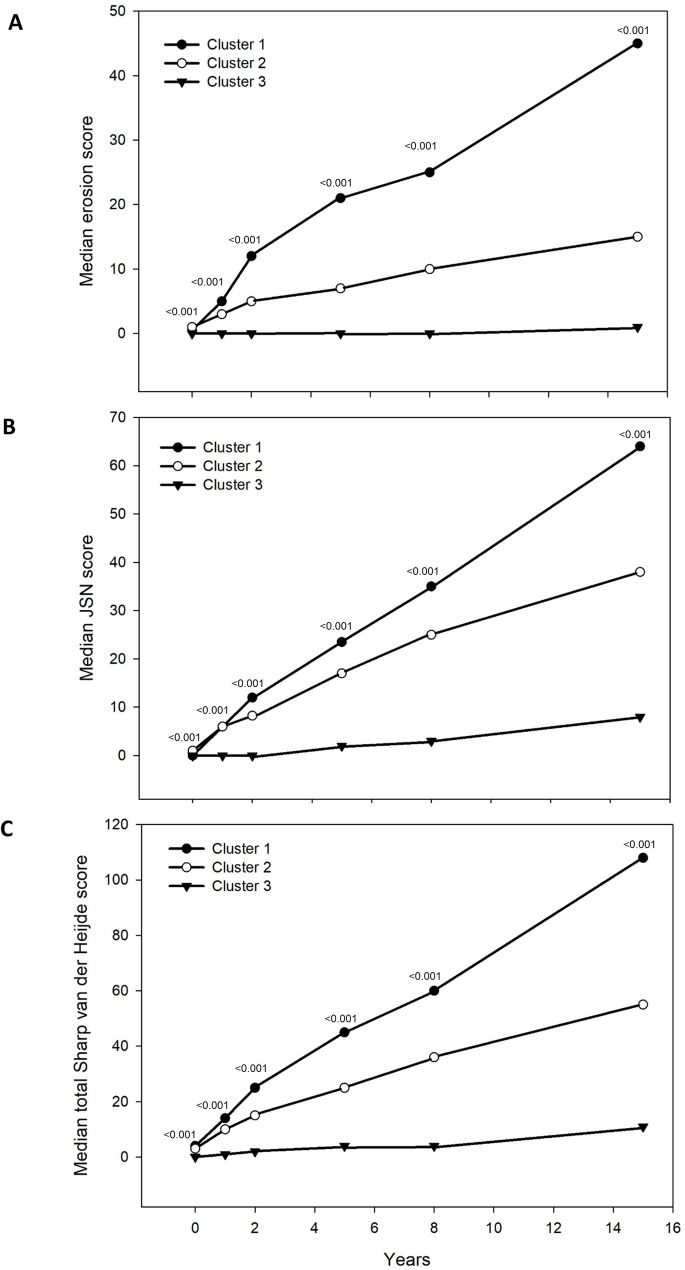
Radiographic progression in the three different clusters over 15 years. Median erosion score (ES), panel **A**, median joint space narrowing score (JSN), panel **B**, and median total Sharp van der Heijde score (SHS), panel **C**. The p-values shown are overall values

The radiographic progression for the 990 patients with radiographs at 15 years, is shown in Fig. 1. Already at baseline there were significant differences between cluster 1 vs. 3 and 2 vs. 3 regarding both ES and JSN, Table 1.

The radiographic progression was most pronounced in cluster 1 over the whole follow-up period, as was rapid progression, both regarding erosions and JSN. All patients in cluster 1 had a progression of 3 or more per year over 15 years, and 93% had a progression of 5 or more per year. At 15 years 29% of the 990 patients did not have any erosions and 68% of them had been erosion-free at all follow-ups.

**Fig. 2:**
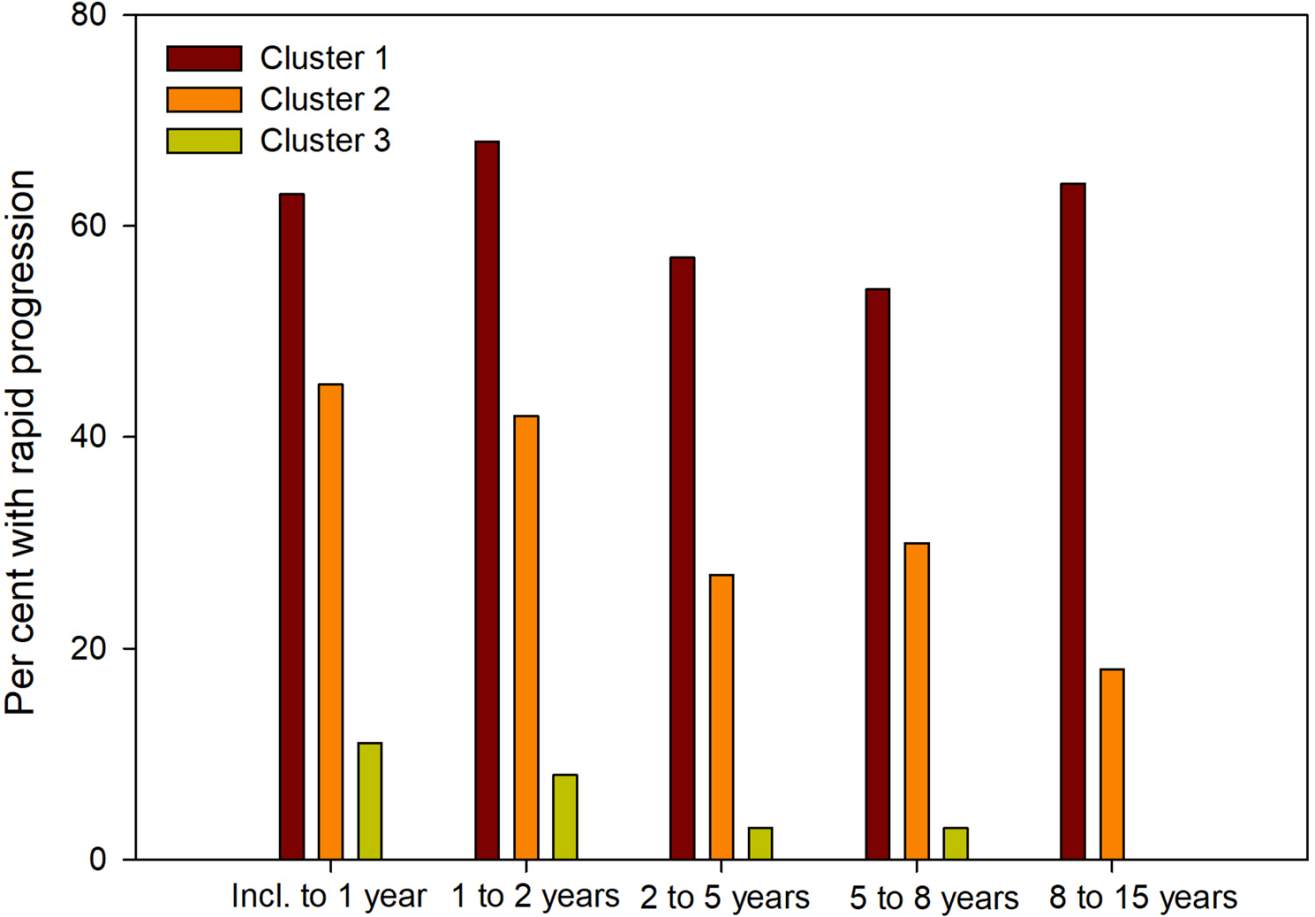
Percentage of individuals with rapid radiographic progression (RRP), increase of Sharp van der Heijde score (SHS) ≥ 5 units per year, within the three clusters

Rapid radiographic progression with SHS ≥ 5 units per year (RRP) is presented in Fig. 2. The differences between the clusters were significant for all three measurements (total SHS, ES and JSN) from inclusion to the different follow-up times, as well as between the different time-points. Cluster 1 shows the worst outcome in all assessments, and cluster 3 the best.

### Clinical outcome in the clusters over 15 years

**Fig. 3:**
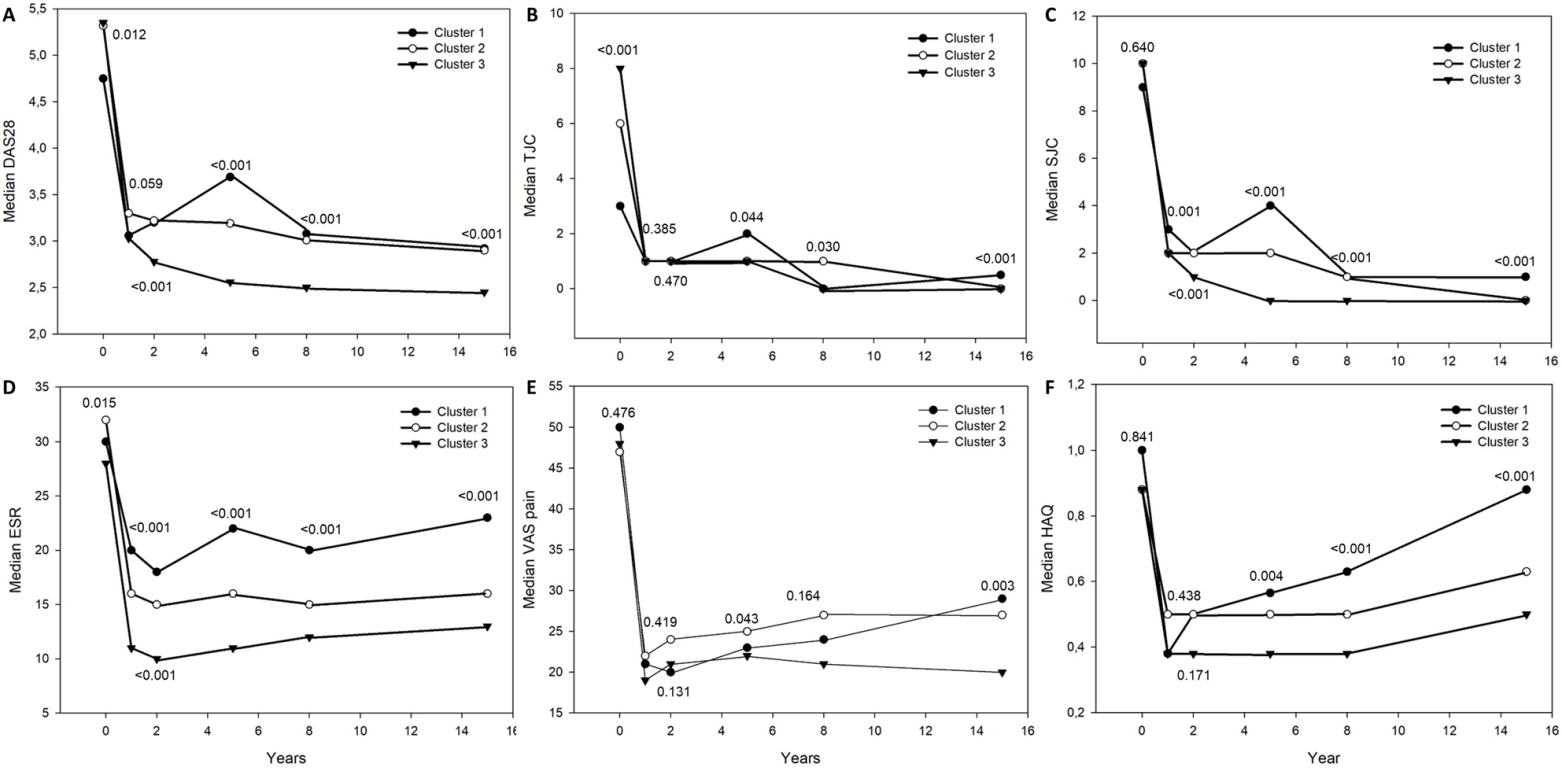
Shows clinical data over 15 years in the three clusters. Panel **A** median 28 joints-disease activity score (DAS28), panel **B** median tender joint count TJC, (28-joints), panel **C** swollen joint count SJC, (28-joints), panel **D** median erythrocyte sedimentation rate (ESR), panel **E** median visual analogue scale (VAS) pain, panel **F**, median health assessment questionnaire (HAQ). P-values are overall values

Figure 3 shows the clinical outcome over time for the three clusters. At baseline cluster 2 differed from cluster 1, having higher DAS28, more tender joints, and were more often started on DMARD-medication. In relation to cluster 3, cluster 2 had at baseline higher SHS, higher ESR and were less often started on DMARD medication. At follow-up, DAS28 and its components SJC and ESR were in cluster 1 and 2 significantly worse compared with cluster 3 at 2, 5, 8 and 15 years, and SJC and ESR also at one year, whereas TJC was worse only at 15 years in cluster 1. From 2 years onwards, a DAS28 ≤ 3.2 was seen in 13% of the patients in cluster 1, 23% in cluster 2 and 28% in cluster 3.

As to HAQ, cluster 2 was worse than cluster 3 at 5 years and both cluster 1 and 2 were worse than cluster 3 at both 8 and 15 years, but as to pain only cluster 2 was worse than cluster 3 at 5 and 15 years.

### Treatment

**Fig. 4:**
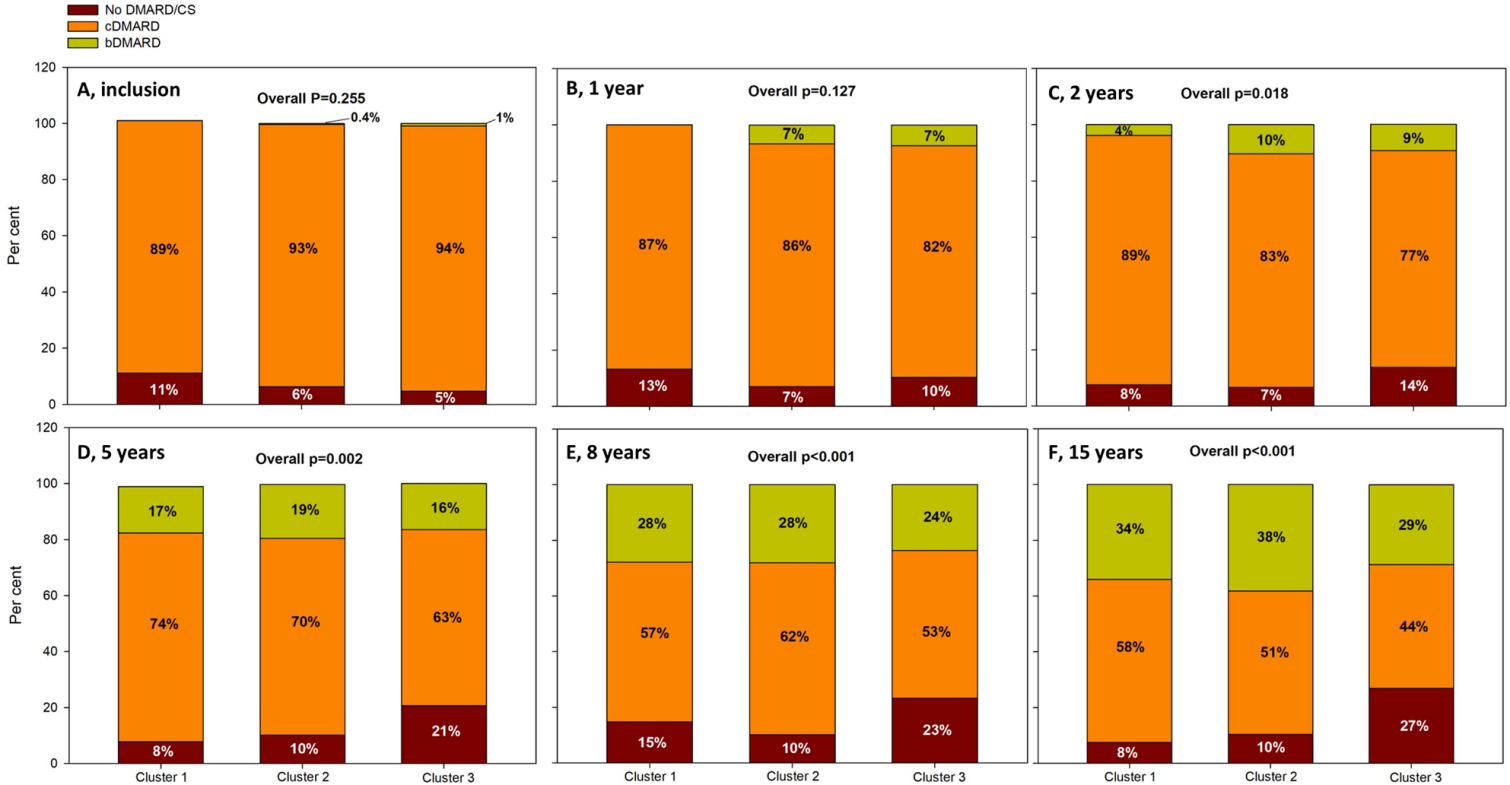
Medical treatment in the three clusters over 15 years. Conventional DMARD (cDMARD) include patients only treated with glucocorticoids (GC) The major part of cDMARD was methotrexate (MTX). The majority of biological disease-modifying antirheumatic drugs (bDMARDs) were TNF inhibitors. Both cDMARD and bDMARD are with or without GC. Panel **A** shows treatment started in the separate clusters at baseline, Panel **B** shows treatment at 1-year follow-up, Panel **C** shows treatment at 2 years, Panel **D** shows treatment at 5 years, Panel **E** shows treatment at 8 years, and Panel **F** shows treatment at 15 years

Methotrexate (MTX) was the most frequently used cDMARD, and tumor necrosis factor (TNF) inhibitors were the most prescribed bDMARDs across all follow-up time points. There were no significant differences in treatment patterns between the clusters at baseline or at the one-year-follow-up. However, from the two-year follow-up onward, a higher proportion of patients in cluster 3 were untreated. Over time, there has been an increased use of bDMARD in all clusters, Fig. 4.

A few of the patients included in the cluster analysis were not treated with any DMARD at the follow-ups. In cluster 1, one individual (2%), in cluster 2, one individual (0.4%) and in cluster 3, 15 individuals (2%).

### Prediction of belonging to cluster 1 or 2

To belong to cluster 1, seropositivity and less tender joints at inclusion were predictive, whereas older age, female sex, seropositivity, and erosions at baseline predicted belonging to cluster 2, Table 2. The univariate model is presented in Table S1. Additionally, we performed sensitivity analyses adjusting for treatment variables (e.g., DMARD/biologic use), which yielded similar results, data not shown.

**Table 2:** Multivariate backward stepwise nominal regression model with cluster 1 and 2 as dependent variables. Cluster 3 is the reference. The table shows the included variables in the final model, age, sex, seropositivity, tender joint count and erosion score at inclusion

	Cluster 2			Cluster 1			
	OR	95% CI	*p*-value	OR	95% CI	*p*-value	
Age at inclusion, year	1.024	1.011–1.037	< 0.001	1.025	0.999–1.051	0.060	
Sex, female	1.534	1.068–2.201	0.020				
Seropositive	2.663	1.786–3.971	< 0.001	6.937	2.086–23.070	0.002	
TJC (28-joints) at inclusion	0.976	0.950–1.003	0.080	0.860	0.797–0.928	< 0.001	
Erosion score at inclusion	1.082	1.037–1.128	< 0.001				

Examples of predicted probabilities of belonging to cluster 1 are as follows. For a 50-year-old seronegative individual with no tender joints and no erosions at inclusion, the probability is 3%. Increasing the age to 70 years raises the predicted probability to 5%.

For a 50-year-old seropositive individual with no tender joints, the predicted probability is 19%, whereas the presence of 10 tender joints reduces the probability to 6%.

For cluster 2, a 50-year-old seronegative individual with no tender joints and no erosions at inclusion, the probability of belonging to cluster 2 is 11% in males and 16% in females. Increasing the age to 70 years raises this probability to 17% in males and 24% in females.

For a 50-year-old seropositive individual with no tender joints and no erosions at inclusion, the predicted probability increases to 26% in males and 35% in females. If the number of tender joints is 10, the probability changes to 21% in males and 29% in females.

Finally, for a 70-year-old seropositive individual with no tender joints and an erosion score of 10 at inclusion, the probability further increases to 55% in males and 65% in females.

## Discussion

The aims of this study were to investigate the radiographic progression over 15 years for erosions and JSN, and the predictive factors for joint damage in patients with early RA. We used the method K-means clustering, an algorithm which groups the items with certain features into different clusters, to identify groups of patients with different radiographic outcomes.

K-means clustering is widely used for data grouping across various research fields due to its efficiency, scalability, and ease of interpretation. In this study, the optimal number of clusters was set to three, which provided the most distinct separation of the radiographic outcomes [24, 26].

We found three distinct groups, where cluster 1 had the worst radiographic outcome, and cluster 3 the best. There were differences between the clusters in the radiographic progression from baseline to all time points for follow-up as well as between the different time points (e.g. 1 year to 2 years, 2 to 5 years and so on). Radiographic over time data, shown in Fig. 1, showed at inclusion SHS 4.0 in cluster 1, 3.0 for cluster 2 and 0 for cluster 3. Corresponding figures at 15 years were 108, 55 and 11 respectively. The patients in cluster 1 were most often seropositive (94%), compared with cluster 2 (84%) and cluster 3 (64%) and this influenced the radiographic outcome, especially erosion progression which is illustrated in Figs. 1 and 2. Seropositivity was an independent predictor to pertain to both cluster 1 and 2.

In the literature, RA associated autoantibodies are consistently associated with worse radiographical outcomes [27, 28], which has been reported in both short-term [29, 30] and long-term [31, 32] studies and has also been shown to be a significant predictor for this outcome.

In a 10-year follow-up study of early rheumatoid arthritis Courvoisier et al. found that baseline radiographic score, ESR and ACPA were the best predictive factors [31]. However, in the present study erosion score at baseline was predictive for radiographic outcome only in cluster 2, probably dependent on too few patients in cluster 1 to reach enough power. Although significant differences between the clusters in ESR at baseline it did not fall out as a predictor for belonging to any of the clusters.

We have previously shown that most erosions appear within two years from symptom onset [33, 34], which is in agreement with the results in the present study, for all three clusters. Also, Lindqvist et al. concluded - in a 10-year study- that joint damage in hands and feet developed early, and progression was most rapid during the first years of disease [35]. Interestingly, though, we found that 20% of the patients were erosion-free during the whole follow-up, which agrees with the results of a study by Svensson et al. who found 24% of 608 patients to be erosion-free over eight years [34].

In cluster 1, the one doing worst, more patients were included in the 1990ths than in the other clusters and they were more often seropositive. From 2000 the bDMARDs were introduced, and at two years the patients in cluster 1 began to be treated with bDMARDs and at five years 17% of them had bDMARDs. The effect can be seen in Fig. 1, especially for ES where we noticed a reduced progression rate after 5 years, most likely attributed to the more widely use of bDMARDs. We registered the same effect on DAS28, SJC, TJC and ESR which improved after 5 years. This trend was seen also at 8 and 15 years. In spite of more treatment the radiographic progression was not halted in cluster 1.

The high TJC in cluster 3 compared with cluster 1 at baseline might explain the high DAS28 in cluster 3 and the fact that there was no difference in medical treatment between the clusters during the first year.

HAQ was higher, that is worse, at 8 and 15 years in cluster 1 and 2 compared with cluster 3. This corresponds to the radiographic progression with worse outcome in cluster 1 and 2 as well as with the higher DAS28 in cluster 1 and 2 throughout the 15 years. Eberhard et al. also showed, in a study of 233 patients with early RA over 5 years, that HAQ was associated with erosions at inclusion, 2 and 5 years as well as with disease activity [36].

Today, with MRI and ultrasound available, the relevance of conventional radiography imaging in the diagnosis and to determine prognosis in rheumatoid arthritis is disputed. In a recently published study Walter et al. conclude that serial follow-up imaging plays an important role in confirming and tracking therapeutic efficacy and prognosis of the treatment and that radiography likely will contribute to diagnosis and management of RA also in the future [1]. In another recently published imaging study with MRI, Dumoulin et al. concluded that the majority of MRI-detected erosions in clinically suspect arthralgia patients did not correspond with radiographic erosive disease or progression and should be regarded with caution to avoid overinterpretation [37]. The use of ultrasound for detection of synovitis and tenosynovitis has evolved during the last decades and is today helpful to early recognize these features which precede the development of erosions.

Ongoing research will show which imaging method is best suited for diagnosis and prognosis, but as we have shown here, conventional radiography still has a place for following the progress of joint damage in RA.

A strength of this study is the large number of patients from a well-controlled cohort of patients with early RA followed prospectively long-term with a structured protocol including radiographs. Therefore, selection bias is not a major issue in this study, and the results could be generalized to patients with RA seen in clinical practice.

The unequal cluster sizes observed in this study are expected, as K-means clustering identifies groups based on similarity and minimizes within-cluster variance rather than enforcing equal group sizes. Consequently, larger clusters likely reflect more common patient profiles, whereas smaller clusters may represent less frequent or more distinct disease phenotypes.

In our cohort, cluster 1 was smaller and characterised by more severe radiographic progression. This could indicate a subgroup of patients with a more aggressive disease course. However, this cluster also included a higher proportion of patients recruited during earlier time periods, when treatment strategies were less intense. Although we adjusted for the inclusion period in the analyses, residual confounding due to evolving treatment paradigms cannot be fully excluded.

Importantly, the identified clusters were stable across sensitivity analyses, supporting the robustness of the clustering approach. Nevertheless, findings related to smaller clusters should be interpreted with some caution.

### Conclusions

Seropositivity was the only common predictor for radiographic progression over 15 years in cluster 1 and 2. For cluster 1 also TJC and for cluster 2 also age, sex and erosion score at baseline predicted cluster belonging. The presence of distinct groups with different patterns of progression in patients with rheumatoid arthritis suggests that patients have different therapeutic needs and that individual treatment strategies are needed.

## Supplementary Information

Below is the link to the electronic supplementary material.

Supplementary Material 1: Figure S1. The scatter plot shows the distribution of patients across the three clusters identified using k-means clustering. The clusters are based on changes in erosion score (ES) and joint space narrowing (JSN) score from baseline to 15-year follow-up. Each dot represents an individual, with colours indicating cluster assignment. The figure illustrates distinct patterns of radiographic progression between clusters.

Supplementary Material 2

## Data Availability

The data analysed are available on reasonable request. All data relevant to the study are included in the article.

## Abbreviations

ACPA: Antibodies to Cyclic Citrullinated Peptides
ACR: American College of Rheumatology
BARFOT: Better Anti Rheumatic FarmacO Therapy
bDMARD: biologic DMARDs
cDMARD: conventional DMARD
DMARD: Disease Modifying Anti-Rheumatic Drug
DAS28: Disease Activity Score 28
ES: erosion score
ESR: Erythrocyte sedimentation rate
GC: glucocorticoids
HAQ: Health Assessment Questionnaire
JSN: joint space narrowing score
MTX: methotrexate
PatGA: patient’s global assessment of health
RA: Rheumatoid arthritis
RF: Rheumatoid Factor
RRP: Rapid Radiographic Progression
SHS: Sharp van der Heijde score
SJC: Swollen Joints Count
TJC: Tender Joint Count
TNF: tumor necrosis factor
VAS: Visual Analogue Scale
