# Supplementary figures and images for: Radiographic outcome over 15 years in patients with early rheumatoid arthritis – a BARFOT-study
Source: BMC Rheumatol. 2026 May 27;10:46. doi: 10.1186/s41927-026-00660-w (PMC13220378; doi:10.1186/s41927-026-00660-w)

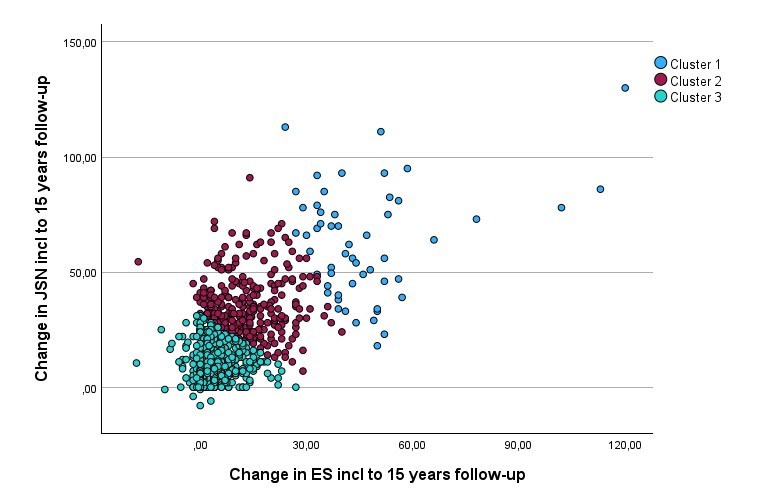

Supplement: Supplementary file 1 — Supplementary Material 1: Figure S1. The scatter plot shows the distribution of patients across the three clusters identified using k-means clustering. The clusters are based on changes in erosion score (ES) and joint space narrowing (JSN) score from baseline to 15-year follow-up. Each dot represents an individual, with colours indicating cluster assignment. The figure illustrates distinct patterns of radiographic progression between clusters. [file 41927_2026_660_MOESM1_ESM.jpg]
