# Supplementary material for: Radiographic outcome over 15 years in patients with early rheumatoid arthritis – a BARFOT-study
Source: BMC Rheumatol. 2026 May 27;10:46. doi: 10.1186/s41927-026-00660-w (PMC13220378; doi:10.1186/s41927-026-00660-w)
Supplement: Supplementary file 2 — Supplementary Material 2 [file 41927_2026_660_MOESM2_ESM.docx]

**Table S1.** Univariate multinominal regression model with cluster 1 and 2 as dependent variables. Cluster 3 is the reference.

|  | Cluster 2 | | | Cluster 1 | | |  |
| --- | --- | --- | --- | --- | --- | --- | --- |
|  | OR | 95% CI | p-value | OR | 95% CI | p-value | |
| Age at inclusion, year | 1.024 | 1.012-1.035 | <0.001 | 1.027 | 1.004-1.050 | 0.020 | |
| Sex, female | 1.314 | 0.948-1.821 | 0.102 | 1.127 | 0.608-2.090 | 0.705 | |
| Smoking, ever | 1.388 | 0.773-2.495 | 0.272 | 1.152 | 0.858-1.547 | 0.346 | |
| RF-positive | 2.408 | 1.734-3.344 | <0.001 | 3.570 | 1.717-7.421 | <0.001 | |
| ACPA positive | 3.237 | 2.171-4.827 | <0.001 | 4.420 | 1.824-10.709 | <0.001 | |
| Seropositive | 2.886 | 2.002-4.162 | <0.001 | 9.540 | 2.946-30.891 | <0.001 | |
| Included in 1990s | 1.011 | 0.758-1.349 | 0.941 | 1.800 | 1.025-3.163 | 0.041 | |
| DAS28 at inclusion | 0.947 | 0.838-1.069 | 0.376 | 0.729 | 0.578-0.912 | 0.006 | |
| TJC (28-joints) at inclusion | 0.960 | 0.935-0.985 | 0.002 | 0.840 | 0.780-0.905 | <0.001 | |
| SJC (28-joints) at inclusion | 0.992 | 0.966-1.018 | 0.537 | 0.978 | 0.927-1.032 | 0.422 | |
| ESR at inclusion, mm/h | 1.007 | 1.001-1.013 | 0.024 | 1.007 | 0.996-1.019 | 0.214 | |
| VAS pain at inclusion, 0-100 | 0.997 | 0.991-1.003 | 0.282 | 0.995 | 0.983-1.007 | 0.406 | |
| VAS PatGA at inclusion, 0-100 | 0.998 | 0.992-1.004 | 0.475 | 0.994 | 0.983-1.005 | 0.259 | |
| HAQ at inclusion, 0-3 | 0.906 | 0.714-1.149 | 0.415 | 0.807 | 0.505-1.288 | 0.369 | |
| Erosion score at inclusion | 1.104 | 1.059-1.151 | <0.001 | 1.059 | 1.003-1.118 | 0.039 | |
| Treatment at inclusion |  |  |  |  |  |  | |
| cDMARD or bDMARD incl | 1 |  |  |  |  |  | |
| No DMARD incl | 1.335 | 0.719-2.476 | 0.360 | 2.523 | 1.006-6.332 | 0.049 | |

ACPA, Anti-citrullinated protein antibodies; bDMARD, biological disease modifying anti-rheumatic drug; cDMARD, conventional DMARD; DAS28, 28 joints-disease activity score; ES, erosion score; ESR, erythrocyte sedimentation rate; HAQ, health assessment questionnaire; PatGA, patient global assessment; RF, rheumatoid factor; Seropos, ACPA and/or RF positive; SJC, 28-joints swollen joint count; TJC, 28-joints tender joint count, VAS; Visual Analogue Scale

.
